# Relationship Between Amyloid Beta Deposition and Gene Expression Patterns in Alzheimer's Disease

**DOI:** 10.1002/alz70855_100138

**Published:** 2025-12-23

**Authors:** Junyin Lu, Ling Xiao, Xuewen Xiao, Yongxiang Tang, Bin Jiao, Lu Shen

**Affiliations:** ^1^ Xiangya Hospital, Central South University, Changsha, Hunan, China

## Abstract

**Background:**

AD is a neurodegenerative condition characterized by progressive neuronal loss and the abnormal deposition of Aβ proteins. To date, the in vivo Aβ deposition measured by ^11^C‐PIB PET and the underlying gene expression patterns in patients with AD remain unclear.

**Method:**

In this study, we consecutively collected ^11^C‐PIB PET scans from AD patients (*n* =  151) and controls (*n* =  76) between 2018 and 2024. We identified the transcriptional profiles of TLE risk genes across the brain through gene expression analyses conducted on six postmortem donors from the Allen Human Brain Atlas. By integrating the neuroimag‐ing and transcriptomic data, we examined the relationship between the expression of AD‐associated genes and Aβ deposition in AD. Furthermore, we performed functional enrichment analyses on the genes with higher weight in partial least squares regression using Metascape. Next, we analyzed the interaction between the enriched genes using STRING (https://cn.string‐db.org/), which returned the gene‐gene network based on genetic interaction.

**Result:**

We observed extensive deposition of Aβ proteins in the cortical regions of AD patients, primarily in the temporal‐parietal and frontal‐parietal cortices. Furthermore, the pattern of Aβ deposits were spatially associated with the expression of AD risk genes throughout the brain (*r* = 0.759, *p* < 0.001). In particular, we identified 223 downregulated genes, which are predominantly enriched in the Notch signaling pathway and pathways related to neuronal differentiation. Among these AD risk genes, downregulated genes such as EGFR, RHOA, VASP, RDX, and MSN may play a critical role in the pathogenesis of AD.

**Conclusion:**

Our study provides evidence that the spatial expression patterns of downregulated risk genes underlie the in vivo Aβ deposition patterns in AD. These imaging‐transcriptomic findings have the potential to guide the development of molecular and genetic network‐based therapeutic approaches for AD.

